# Recurrent prurigo nodularis related to infected tonsils: a case report

**DOI:** 10.1186/1752-1947-2-243

**Published:** 2008-07-24

**Authors:** Michael Katotomichelakis, Dimitrios G Balatsouras, Konstantinos Bassioukas, Nikolaos Kontogiannis, Konstantinos Simopoulos, Vassilios Danielides

**Affiliations:** 1 Department of Otolaryngology, Medical School, Democritus University of Thrace, Greece; 2 Department of Otolaryngology, Tzanion General Hospital of Piraeus, Greece; 3 Department of Dermatology, Medical School, University of Ioannina, Greece; 4 Second Department of Surgery, Medical School, Democritus University of Thrace, Greece

## Abstract

**Introduction:**

Prurigo nodularis is an unusual disorder of unknown aetiology, which is notoriously resistant to therapy, and is characterized by extremely pruritic nodules with well-defined clinical symptoms and histopathological findings.

**Case presentation:**

We report the case of a patient presenting with pruritic papules and nodules on his legs, arms and trunk over the past 4 years, recurring after episodes of acute tonsillitis. Although oral and topical corticosteroids, oral antibiotics and emollients were used in his therapy, only tonsillectomy finally proved the definitive treatment.

**Conclusion:**

We discuss the aetiopathogenesis, diagnosis and treatment of prurigo nodularis associated with chronic tonsillitis, and we further review the literature on this rare condition.

## Introduction

'Prurigo' is a widely used term without a precise definition. There are three clinical types: acute, subacute and chronic [1]. The chronic form includes prurigo nodularis (PN) of Hyde. This is an unusual disorder of unknown aetiology characterized by extremely pruritic nodules and with well-defined clinical symptoms and histopathological findings. Its aetiology is related to atopic, neuronal, traumatic, metabolic and other factors [1-3]. PN is notoriously resistant to therapy [4].

In this case report, we present the first, to the best of the authors' knowledge, reported case of recurrent PN clinically related to infected tonsils. We focus on its pathogenesis and treatment.

## Case presentation

**Figure 1:**
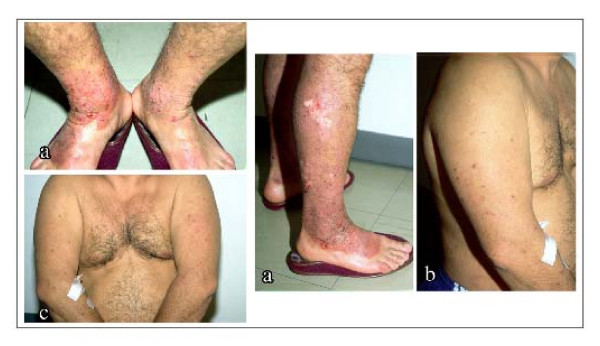
**Pruritic papules and scattered nodules**. Pruritic papules and scattered nodules can be seen (a) on the legs, (b) on the arms and (c) on the trunk of our patient.

A 42-year-old man visited our outpatient dermatology clinic with papulonodular, pruriginous eruption on the limbs. Clinical examination revealed grouped and scattered pruritic papules and nodules on his legs, arms and trunk (Figure 1).

Four years earlier, the patient had been in a car accident and the first papules had appeared around the trauma and burn scars. Gradually, they disseminated to the legs, the arms and the chest. He also had irritant contact dermatitis on the hands, possibly from using detergents. The patient also reported allergic rhinitis, conjunctivitis and repeated episodes of tonsillitis over the last 5 years.

Results of haematological and biochemical examinations were within normal limits, as were the rapid plasma reagin, anti-HIV, C-reactive protein and serum IgE results. There was a raised antistreptolysin O titre (ASTO) of 800 IU/ml. His chest X-ray and his urine test were also normal. A skin biopsy from the trunk revealed a pseudo-epitheliomatous acanthosis, hyperkeratosis and vascular hyperplasia of the upper dermis with a mild inflammatory perivascular infiltration, a scenario compatible with PN. Characteristics of a specific inflammation were not observed.

The patient was treated with oral methylprednisolone 16 mg gradually tapered, oral antibiotics, hydroxyzine 25 mg, local clobetasol propionate 0.05% cream and emollients. This treatment led to a regression for some time, but later the same clinical symptoms recurred.

One month after regression of the nodules, the patient underwent patch testing with European standard battery and metals (TROLAB^®^), following the International Contact Dermatitis Research Group guidelines [5]. The reactions were negative at 48 and 96 hours and at 7 days. The same tests were repeated 2 months later and were again negative. The patient had also been subjected to skin prick testing and radioallergosorbent tests for the detection of aeroallergens implicated for allergic rhinitis, 2 months before consultation. Since these tests were negative and as there were no current clinical or endoscopic signs of rhinitis, we decided to not repeat allergic testing for rhinitis.

**Figure 2:**
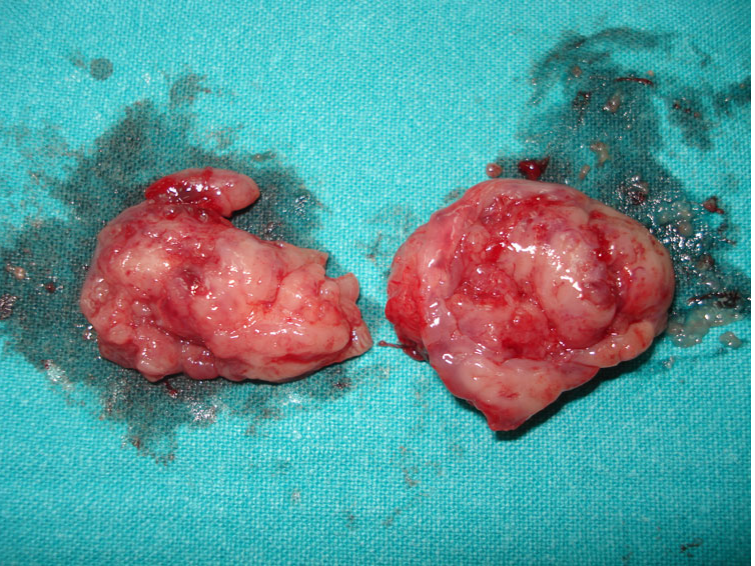
The removed tonsils of our patient.

The patient's history with chronic tonsillitis in relation to the high ASTO levels led us to believe that tonsillitis could be a possible cause for PN, and the patient underwent a tonsillectomy (Figure 2). The nodules started to regress gradually with the application of local steroids. Six months later, they had totally disappeared; only the scars from the car accident and some hyper-pigmentation were apparent. In 6 years of follow-up, the patient is doing well with no skin lesions and with normal ASTO levels.

## Discussion

Chronic, intensely itchy nodules clinically characterize PN. PN appears mainly in adults of both sexes aged 20 to 60 years and especially in middle-aged women [1,4], although cases affecting children have also been described. The characteristic lesions are hard pruriginous nodules, round and keratotic, 1 to 3 cm in diameter with a raised, warty surface. The early lesions are red and may show a variable urticarial component, but they tend to be pigmented. Crusts and scales may cover recently excoriated lesions. They are usually grouped and may vary greatly in number [3]. There is a tendency for symmetrical distribution, with predominance on the extensor surface of limbs [1,6]. Case reports record that nodules also appear on the trunk, and no part of the body is exempted [6]. Lichenoid plaques are also a frequent finding [3].

In our patient, pruritic papules and scattered nodules were observed symmetrically on the legs, the arms and the chest. Patients are tormented with crises of pruritus of intense severity. New nodules develop from time to time, and existing nodules may remain pruritic indefinitely, although some may regress spontaneously and leave scars. In most cases, the disease runs a very protracted course with exacerbations and remissions, as in our case.

The aetiology of PN is still unknown. It has been reported in relation with atopy in 65% to 80% of cases, but other studies [7] suggest not only metabolic causes such as anaemia, hepatic dysfunction, uraemia and myxoedema, and focal causes such as venous stasis, folliculitis and nummular eczema, but also psychosocial disorders [3]. Psychogenic factors, such as emotional stress, depression or anxiety, should be considered in all cases. Although there was no evidence of a psychological cause in our patient, this cannot be ruled out as a contributing factor, owing to the long duration of PN. Important external causes of prurigo include heat, cold, light, insect bites, ectoparasites and allergenic contactants of the skin, as well as food and drug allergies [8]. Our patient mentioned an atopic diathesis that manifested with allergic rhinitis and conjunctivitis. He also had hand dermatitis, probably caused by irritants, as his history was compatible with the exposure of his atopic dry skin to detergents. Allergens may have been a cause of his dermatitis, but patch tests, at least with European standard battery and metals, were negative twice.

Other important aetiological factors include internal infections, such as intestinal parasites, echinococcosis and internal foci of infection such as colitis or infected tonsils [8]. It is well known that superantigens from bacterial foci can cause many different skin reactions [8]. Our patient had a history of chronic tonsillitis with raised ASTO (800 IU/ml) and clinical worsening of PN followed exacerbations of tonsillitis with fever and weakness. This suggests that streptococci might be the main aetiological factor of the disease. Malignant lymphomas, malignant tumours, solid tumours, carcinoid syndrome, polycythaemia, obstructive biliary disease, chronic renal failure, rubra vera, hypothyroidism and hyperthyroidism, diabetes mellitus, obesity, hypertension, peptic ulcer, alcoholism, sarcoidosis, psoriasis, Gilbert's disease, folliculitis or pityriasis capitis, gluten enteropathy and other forms of malabsorption are other aetiological factors [3,7], as well as acquired immunodeficiency syndrome [9]. All of these factors were excluded in our patient. Endocrine factors, such as ovarian dysfunction, or traumatic, mycobacterial or *Staphylococcus aureus *[8] or neuronal factors [10] (where Merkel cells are increased in number suggesting a neurocutaneous abnormality) are other possible causes of PN that were not present in our case.

Microscopically, the findings include large, irregular or even pseudo-epitheliomatous cells, acanthosis, hyperkeratosis and parakeratosis, with oedema in the lower epidermis and upper dermis, and also an inflammatory perivascular infiltrate in the upper dermis [1,4]. We observed all of these findings in the skin biopsy.

Treatment of prurigo is symptomatic and determined on a case-by-case basis. At the outset, it includes general measures such as trimming the fingernails, avoiding scratching and hospitalization for better observation [8]. Topical agents recommended include emollients and corticosteroids combined with lactic or retinoic acid to enhance penetration, menthol, tar and occlusion with bandages (with or without steroids) [1,4,8]. Intralesional corticosteroids [1,4,8], such as dexamethasone or triamcinolone, are far more effective but should be used with care to avoid side effects. Sedatives and tranquilizers or antihistamines [1] are of great help. Antibiotic therapy (erythromycin, clofazimine for 6 months) is also of great importance [4] and thalidomide is considered an effective treatment [1,4]. Localized phototherapy, photochemotherapy applied topically and nitrogen cryotherapy [8] are also included in the treatment of PN. The number of simultaneously treated nodules and the duration of cryotherapy for individual nodules must be determined in each case. Benoxaprofen, cyclosporin, azathioprine and topical capsaicin have also been used with success in some cases [11].

Spontaneous regression is rare and relapse is common, despite the availability of several therapeutic options. In our case, oral antibiotics, oral hydroxyzine 25 mg daily and oral prednisolone 16 mg tapered gradually, together with local clobetasol propionate 0.05% cream and emollients, were used with good results in all treatment courses, but there were relapses soon after. Tonsillectomy was the final and definitive treatment of PN in our patient, as may be evidenced from the history of our patient and the follow-up of the disease. Pre-operatively, we could not prove that chronic tonsillitis was the cause of the skin disease. Nevertheless, tonsillectomy was indicated owing to the chronic infection in conjunction with elevated ASTO. Eradication of the streptococcal foci was obtained by tonsillectomy and the ASTO was decreased, resulting in the disappearance of the lesions.

Therefore, it may be safely concluded that streptococcus was at least one of the causes of the disease, and possibly the only cause. Other possible causes or aggravating factors of the skin disease may have included atopy, emotional stress and the car accident that our patient experienced prior to the initial clinical manifestations.

## Conclusion

We have reported the case of a patient with PN, clinically strongly related to chronic tonsillitis with exacerbations and remissions, who was finally successfully cured by tonsillectomy. Atopic diathesis and possible emotional stress may have been background factors but were not the main aetiology. To the best of the authors' knowledge, after the first mention of a probable relation between tonsillitis and PN by Drake [2] and a general description of chronic tonsillitis as a cause of PN by Arnold et al. [8], this is the first reported case of a documented clinical relationship between PN and tonsillitis.

## Abbreviations

ASTO: antistreptolysin O titre; PN: prurigo nodularis.

## Competing interests

The authors declare that they have no competing interests.

## Authors' contributions

MK examined the patient and participated in the design of the study and the drafting of the manuscript. DGB participated in the design of the study and the drafting of the manuscript. KB conceived of the study, acquired the data and critically reviewed the manuscript. NK conceived of the study and examined the patient. KS participated in the design of the study and critically reviewed the manuscript. VD conceived of the study, examined the patient and critically reviewed the manuscript. All authors read and approved the final manuscript.

## Consent

Written informed consent was obtained from the patient for publication of this case report and any accompanying images. A copy of the written consent is available for review by the Editor-in-Chief of this journal.
